# Assessment of glucocorticoid and antibiotic exposure as risk factors for diabetes mellitus in selected dog breeds attending UK primary‐care clinics

**DOI:** 10.1002/vetr.2785

**Published:** 2023-04-02

**Authors:** Angela M. Heeley, Dave C. Brodbelt, Dan G. O'Neill, David B. Church, Lucy J. Davison

**Affiliations:** ^1^ Department of Pathobiology and Population Sciences Royal Veterinary College Hatfield UK; ^2^ Department of Clinical Science and Services Royal Veterinary College Hatfield UK

**Keywords:** antibiotics, case–control study, diabetes mellitus, glucocorticoids, risk factors, VetCompass

## Abstract

**Background:**

Diabetes mellitus (DM) is an important endocrine disorder in dogs. This study explored prior exposure to glucocorticoids or antibiotic treatment as risk factors for developing DM in dogs attending primary‐care VetCompass clinics in the UK.

**Methods:**

A breed frequency matched case–control study nested in a cohort of dogs (*n* = 480,469) aged 3 years or over was used to explore associations between glucocorticoid and antibiotic exposure and the odds of developing DM.

**Results:**

A total of 565 cases and 2179 controls were included. Dogs with DM had over four times the odds of exposure to glucocorticoids within 6 weeks prior to diagnosis (odds ratio [OR] 4.07, 95% confidence interval [CI] 2.41–6.89, *p* < 0.001) compared to controls within 6 weeks prior to a randomly selected quasi‐date of diagnosis. Dogs that had only one unique documented antibiotic course had a decreased odds of developing DM (OR 0.65, 95% CI 0.46–0.91, *p* = 0.012) compared to dogs that had no documented courses of antibiotics.

**Limitations:**

This study only included selected breeds, so the results may not be generalisable to all dog breeds.

**Conclusions:**

Exposure to glucocorticoids is associated with a substantial increase in the risk of developing DM for the dog breeds included in this analysis.

## BACKGROUND

Diabetes mellitus (DM) is an endocrine disorder characterised by persistently elevated blood glucose and has an estimated annual prevalence in the general UK dog population of 0.26%–0.32%.^1^, ^2^, ^3^ Clinical signs result from dysfunction or loss of pancreatic islet cells, insulin deficiency and persistent hyperglycaemia and include polyuria, polydipsia, polyphagia and weight loss.^5^, ^6^, ^7^ In humans, DM is classified broadly according to the underlying aetiology, such as type 1 DM (usually immune‐mediated beta‐cell destruction) and type 2 DM (predominantly due to insulin resistance).^8^ In dogs, the aetiology is less well understood, and although there are similarities between some cases of canine DM and type 1 DM in humans, there is conflicting evidence as to whether this is caused by immune‐mediated beta‐cell destruction in dogs.^9^, ^10^, ^11^ The pathogenesis of DM in dogs is complex, and is classified as either insulin‐deficient DM (beta‐cell‐related disorders) or insulin‐resistant DM (target‐organ disorders).^5^ The exact pathogenesis is likely to be heterogenous within the general dog population, involving interactions between both non‐environmental (genetic) and environmental factors,^11^, ^12^, ^13^ resulting in the complete beta‐cell loss and insulin dependence that characterises most cases of canine DM.

Over recent decades, there has been a sharp rise in the incidence of type 1 DM in humans,^14^ and an increase in the reported incidence and prevalence of type 2 DM.^15^, ^16^, ^17^ Although there are limited data for incidence trends for DM over time in dogs, some studies from the US report that the prevalence in dogs has increased from 13.1 cases per 10,000 in 2006 to 23.6 cases per 10,000 in 2015, a rise of 79.9%.^18^, ^19^ The increase in numbers of both people and dogs living with DM (prevalence) is likely to partially reflect improved treatment and survival, but this does not explain the rising numbers of new cases (incidence) reported for human type 1 and type 2 DM. It is suggested that environmental factors, particularly in early life, may be partially responsible for this increase.^20^ Interactions between genetic and environmental factors in diabetes risk are poorly understood, although postulated mechanisms in humans include environmental effects on the immune system in type 1 DM and on pancreatic beta‐cell stress in type 2 DM.

Different environmental factors have been implicated in the development of DM depending on the aetiology. Those associated with the onset of human type 1 DM include older maternal age (39–42 years), pre‐eclampsia, caesarean section delivery, increased birthweight, viruses, dietary factors, air pollution and alterations in gut microbial flora.^21^, ^22^, ^23^, ^24^ Environmental factors associated with the development of human type 2 DM include alterations in gut microbial flora, obesity and low socioeconomic position.^25^, ^26^, ^27^ Environmental factors associated with a type of DM known as medication‐induced DM in humans include prescribed medications such as glucocorticoids, immunosuppressants, antipsychotic medications and calcineurin inhibitors.^28^, ^29^ These medications may induce DM through a variety of mechanisms, including decreased insulin secretion, decreased insulin action and direct neural effects.^28^

Risk factors reportedly associated with the development of DM in dogs include age, sex and breed, polymorphisms in immune response genes, and environmental factors including neutering status, obesity, infection, medication and concurrent disease.^2^, ^3^, ^30^, ^31^, ^32^ In dogs, treatment with glucocorticoids or progesterone has been associated with an increased risk of DM.^2^, ^33^, ^34^, ^35^ Glucocorticoids cause insulin resistance, leading to hyperglycaemia and impaired glucose tolerance, which may result in diabetes if pancreatic beta‐cells become exhausted.^36^, ^37^ Progestogens exert their effects as potent antagonists of insulin and, combined with the effect of growth hormone produced by the canine mammary glands, are the driving factor behind naturally occurring dioestrus‐associated DM.^38^

The gut microbial flora—the microbiome—are resident organisms that live in close association with the intestinal tissues, a mutualistic relationship that can influence host immunity.^39^ Alterations in the composition of the microbiome have been associated with both type 1 DM^40^, ^41^, ^42^ and type 2 DM in humans^26^, ^43^ and recently also with DM in dogs,^44^ but it is unclear whether these relationships are causal or consequential. Understanding which microbial alterations are associated with DM and how these arise is not well understood. Suggested mechanisms by which certain microbiota, such as *Bacteroidetes* spp. may predispose to autoimmune pancreatic islet destruction in type 1 DM include antigen mimicry, disruption of gut epithelial integrity and release of immunomodulatory metabolites.^45^, ^46^ In type 2 DM, suggested mechanisms include microbiome impact on glucose homeostasis.^47^

There are many factors that can influence the composition of the microbiome in humans, including host genetics and environmental factors in early life, such as delivery mode, feeding method, hospitalisation and antibiotic intake.^48^, ^49^ Alterations in the microbiome in response to antibiotic intake contribute to the hypothesis that antibiotic exposure may be a risk factor for the development of DM. However, research exploring the association between antibiotic exposure and the development of DM has had conflicting results. There is contrasting evidence from studies in rodents that antibiotic‐induced alteration of the gut microbiota may either inhibit or may accelerate the development of type 1 DM.^50^, ^51^, ^52^, ^53^, ^54^ Similarly, in humans, although some studies identified associations between the incidence of type 1 DM and the number of antibiotic courses prescribed, the timing of administration and the type of antibiotic prescribed,^55^, ^56^, ^57^, ^58^, ^59^ other studies failed to show any association between antibiotic administration and the incidence of type 1 DM.^60^, ^61^, ^62^, ^63^, ^64^

It is possible that exposure to medications may impact the development of DM in dogs, either in the shorter term, as in the case of glucocorticoids, or in the longer term, as hypothesised by antibiotic influences on the microbiome. To date, there have been limited studies exploring associations between prescribed medications and DM in dogs. However, as both glucocorticoids and antibiotics are regularly prescribed medications in first opinion veterinary practice,^65^, ^66^, ^67^, ^68^, ^69^ a deeper understanding of the effects of these drugs on the development of DM could improve our knowledge of the pathogenesis of the disease.

Consequently, this study aimed to explore associations between prescription of glucocorticoids and antibiotics in first opinion veterinary practice with the odds of developing DM in dogs. Secondary aims were to describe the patterns and types of glucocorticoid and antibiotic prescriptions within the study population. A greater understanding of the impact these drugs may have on disease development could aid primary‐care clinicians in the rational use of glucocorticoids and antibiotics, particularly in assessing the risk or benefit associated with such medications in individual patients with a view to preventing cases of DM.

## METHODS

This project explored electronic patient record (EPR) data collected from UK‐wide primary‐care practices participating in the VetCompass research programme at the Royal Veterinary College (RVC).^70^ These participating practices contribute anonymised clinical data from over 9 million dogs to generate an online research database for large‐scale epidemiological studies. In this project, the study population included all dogs aged 3 years or over on 1 January 2016 under primary veterinary care at a VetCompass practice during 2016. Dogs under veterinary care were defined as those with either (1) at least one EPR (VeNom diagnosis term,^71^ free‐text clinical note, treatment or bodyweight) recorded during 2016 or (2) at least one EPR recorded during both 2015 and 2017.

### Case–control study

A case–control study design was used to explore associations between prior glucocorticoid and antibiotic exposure and the diagnosis of DM. Assuming a 15% exposure to the medication among controls,^65^, ^69^ sample size calculations estimated that approximately 400 cases and 1600 non‐diabetic controls would be required to identify prior medication as a risk factor with an odds ratio (OR) of 1.5 or more (case:control ratio 1:4, 95% confidence interval [CI], 80% power and a 15% exposure among controls, using OpenEpi online calculator, and the methods described by Fleiss et al.).^72^, ^73^ In order to improve the power of the study, a 1:4 case:control ratio was used.^74^ Ethical approval was granted by the RVC Social Science Ethical Review Board (reference number SR2018‐1652).

### Cases

DM cases were identified by screening the EPRs for terms related to DM diagnosis/management within the free‐text clinical records (diab, insul, hyperg, mell, glucose, DM, ketoa, ketou, IDDM, fruct, curve, insuv, prozi, canins, vetp, vet pen), treatment records (canins, insul, prozi, neutral, lent, vetp, vet pen, insuv) and recorded VeNom diagnosis terms^71^ (diabetes mellitus, diabetes mellitus—unstable, diabetes mellitus—stable, diabetic ketoacidosis). The clinical records of all these potential DM cases were read manually to determine inclusion according to the primary case definition of a confirmed veterinary diagnosis of DM, treatment with insulin or strong evidence of a veterinary diagnosis based on hyperglycaemia, glucosuria and appropriate clinical signs. Dogs less than 4 years of age at the time of first diagnosis and dogs treated with insulin for hyperkalaemia without hyperglycaemia were excluded.

The date of first diagnosis (defined as the earliest date of either veterinary confirmation of diagnosis, confirmatory laboratory results or initiation of insulin therapy) and the breed were extracted for all DM cases fulfilling the above case definition. Cases were categorised according to breed and classified as either incident (newly diagnosed in 2016 or 2017) or pre‐existing (first diagnosed with DM prior to 2016). Fourteen breeds containing 10 or more incident cases in 2016 or 2017 were selected for further analysis, including both common UK dog breeds and those at high intrinsic risk of developing DM.^2^, ^3^ This was done in order to increase the precision of the model estimates and model stability, and to include high‐risk breeds that may be more likely to demonstrate an effect of prior glucocorticoid and antibiotic exposure.

### Controls

Control dogs were frequency matched to the 14 breeds with 10 or more cases at a 1:4 case:control ratio. All dogs from the 14 breeds within the underlying study population that were not identified as a DM case according to the primary case definition were eligible as potential controls. These potential control dogs were randomly ordered within each breed group prior to matching using the ‘RAND’ function in Microsoft Excel.^75^

A quasi‐date of diagnosis was determined for each control by selecting the EPR entry closest to a random date in 2016 and 2017 generated using the ‘RANDBETWEEN’ function in Microsoft Excel.^75^ The date of diagnosis for cases and quasi‐date of diagnosis for controls are referred to as the index date from here.

Controls were excluded if they were less than 4 years of age at the index date or if they had evidence in the EPR of a DM diagnosis up to January 2021. The date of final patient–practice interaction was recorded for all controls and used to determine the oldest age at which these dogs were known to not have developed DM.

### Data collection

Demographic data extracted for each case and control included breed, date of birth, insurance status, sex and neuter status at the index date, along with the overall veterinary group (a collection of veterinary clinics under the same ownership) and the individual veterinary practice (clinic ID) that each dog attended. The date of first patient–practice interaction in the available EPR was extracted for all cases and controls and used to determine the length of documented history prior to the index date.

The exposure to glucocorticoid variable was binary and defined as prescription or documented usage of systemic glucocorticoids (prednisolone, dexamethasone sodium phosphate, methylprednisolone or methylprednisolone acetate) within 6 weeks prior to the index date. Topical glucocorticoid usage was excluded. Exposure to glucocorticoids was broken down by the breed frequency matched variable. In addition, the type of glucocorticoid and the indication for use were recorded. These data were extracted for each dog by manually checking the EPR.

A unique antibiotic course was defined as an injection of a long‐acting antibiotic, an injectable antibiotic given on 3 or more consecutive days, or an oral antibiotic prescription. Exclusions included topical medications, a single injection of a short‐acting antibiotic, an antibiotic prescribed within 30 days of a previous antibiotic prescription and any antibiotic prescribed within the 30 days preceding the index date. These data were extracted from the treatment records of each dog. The number (%) of dogs having at least one unique antibiotic course, for all dogs and broken down by the breed frequency matched variable, was reported for descriptive purposes only, as was the median number and range of antibiotic courses per year of documented dog history. The type of antibiotic and the classification according to the World Health Organization (WHO) list of critically important antimicrobials (CIA) for human medicine^76^ were also recorded for descriptive purposes. For data analysis, the antibiotic use variable was defined as the number of unique antibiotic courses to which the animal had been exposed and was categorised as exposure to 0, 1, 2 or 3+ unique courses. Two versions of this variable were created by counting the number of unique antibiotic courses from (1) all the documented history of the dog (total number of unique antibiotic courses documented per dog) and (2) the documented history of the dog in the year preceding the index date only (number of unique antibiotic courses within year prior to index date).

### Data analysis

The data were checked and cleaned in Microsoft Excel before performing statistical analysis using Stata Version 16.1 (Stata Corporation). Two‐year period prevalence and incidence values of DM with 95% CIs were reported for all dogs within the study population, as were sex and the median age. Descriptive statistics characterised exposure to glucocorticoids and antibiotics, age at the index date, breed, sex, sex–neuter status, insurance status and veterinary group for the incident DM case and control dogs separately. Age at the date of the final patient–practice interaction was described by the range and median for control dogs only. Exposure to glucocorticoids and exposure to antibiotics were categorised as described above. Age (years) at the index date was described by the median and range for both cases and controls, and also categorised into four groups (4 to less than 7, 7 to less than 9, 9 to less than 12, 12 or more) crudely corresponding to quartiles of the case ages, consistent with previous studies.^2^, ^3^ The breed variable contained individual breeds with 10 or more incident DM cases recorded. The sex–neuter variable was categorised into seven groups: female entire (FE), female neutered, female unknown neuter status, male entire (ME), male neutered, male unknown neuter status and sex unrecorded. The categorical variables were reported as the number (%) of dogs exposed to that variable or level of variable.

Univariable binary logistic regression and Wilcoxon rank sum testing were used to evaluate associations between the main risk factors of interest (glucocorticoid use 6 weeks prior to index date, total number of unique antibiotic courses documented per dog and number of unique antibiotic courses within year prior to index date) and potential confounders (age at diagnosis, sex, sex–neuter status, insurance status and veterinary practice) and the diagnosis of DM, as appropriate. Univariable analysis was performed on (1) all dogs under analysis and (2) a subset of these dogs that had at least 1 year of documented history.

Variables having an association with the diagnosis of DM with a *p*‐value less than 0.2 in the univariable analysis were taken forward to mixed effects multivariable logistic regression. Both the main factors of interest were included in a single final model rather than two separate models. This is because antibiotics and glucocorticoids are often prescribed together, such as in 34% of canine pyoderma cases,^77^ and therefore, these factors were likely to confound each other. The final model was built using a manual forwards step‐wise selection approach,^78^ retaining the most significant variable at each step until the final model contained all variables that were significant at the *p*‐value less than 0.05 level. Breed was forced into the final mixed effects model as controls were frequency matched to cases on this variable. Biologically plausible interactions between final model variables were evaluated. Clinic ID was added as a random effect to account for variation in prescribing behaviour and diagnostics between clinics. The model parameters were fitted in Stata version 16.1 using maximum likelihood. Goodness of fit was assessed by the Hosmer–Lemeshow statistic (non‐random effect model), visually comparing observed with expected probabilities, and assessing the area under the receiver operating characteristic (ROC) curve.

## RESULTS

The study population consisted of 480,469 dogs aged 3 years or over on 1 January 2016 under primary veterinary care at 881 primary‐care UK‐wide VetCompass practices. The median age of this study population was 6.7 years (interquartile range [IQR] 4.7–9.4 years, range 3.0–20.0 years), and 48.3% (231,535) were female.

Within this study population, there were 1808 dogs aged 4 years and over with DM during 2016–2017, giving a 2‐year period prevalence of 0.38% (95% CI 0.36–0.39). Of these cases, 1121 were pre‐existing (0.23%, 95% CI 0.22–0.25) and 687 were incident, giving a 2‐year incidence risk of 0.14% (95% CI 0.13–0.15). Fourteen breeds had 10 or more DM cases (Table 1). After excluding cases from breeds containing less than 10 DM cases, 565 incident cases fulfilled the criteria for inclusion as cases in the case–control analysis. These were frequency matched to 2179 controls by breed, meaning the case–control analysis included 2744 dogs. Between them, these dogs offered a total of 7962 dog years of documented clinical history available prior to the index date. The median duration of documented history was 1.74 years (IQR 0.50–4.21, range 0–11.32) for cases and 2.16 years (IQR 0.52–4.96, range 0–17.14) for controls.

**TABLE 1:** Descriptive statistics and univariable logistic regression results (*n* = 2744).

Variable	Cases (%), *n* = 565	Controls (%), *n* = 2179	Odds ratio	95% CI	Category *p*‐value	Variable *p*‐value
Total number of unique AB courses documented per dog						
median (range)	0 (0–18)	0 (0–31)				0.910^a^
Total number of unique AB courses documented per dog						
0	343 (60.7%)	1277 (58.6%)	Base			0.004
1	79 (14.0%)	414 (19.0%)	0.71	0.54–0.93	0.013	
2	52 (9.2%)	226 (10.4%)	0.86	0.62–1.18	0.349	
3+	91 (16.1%)	262 (12.0%)	1.29	0.99–1.69	0.059	
Number of unique AB courses within year prior to index date						
median (range)	0 (0–5)	0 (0–3)				0.017^a^
Number of unique AB courses within year prior to index date						
0	508 (89.9%)	2023 (92.8%)	Base			0.031
1	41 (7.3%)	128 (5.9%)	1.28	0.89–1.84	0.191	
2	10 (1.8%)	22 (1.0%)	1.81	0.85–3.85	0.123	
3+	6 (1.1%)	6 (0.3%)	3.98	1.28–12.40	0.017	
Glucocorticoid use 6 weeks prior to index date						
No glucocorticoid use	514 (91.0%)	2121 (97.3%)	Base			<0.001
Glucocorticoid use	51 (9.0%)	58 (2.7%)	3.63	2.46–5.35		
Age at last interaction						
median (range)		10.39 (4.1–21.0)				
Age at diagnosis (years)						
median (range)	10.0 (4–18.0)	8.3 (4.1–21.0)				<0.001^a^
Age at diagnosis						
4 to <7 years	60 (10.6%)	809 (37.1%)	Base			<0.001
7 to <9 years	128 (22.7%)	472 (21.7%)	3.66	2.64–5.07	<0.001	
9 to <12 years	276 (48.9%)	529 (24.3%)	7.03	5.21–9.50	<0.001	
12+ years	101 (17.9%)	369 (16.9%)	3.69	2.62–5.20	<0.001	
Breed matching variable						
Crossbreed	141 (25.0%)	523 (24.0%)	Base			1.00
WHWT	92 (16.3%)	370 (17.0%)	0.92	0.69–1.24		
JRT	57 (10.1%)	224 (10.3%)	0.94	0.67–1.33		
Yorkshire Terrier	49 (8.7%)	189 (8.7%)	0.96	0.67–1.39		
Labrador Retriever	40 (7.1%)	159 (7.3%)	0.93	0.63–1.38		
Border Terrier	31 (5.5%)	123 (5.6%)	0.93	0.60–1.45		
Bichon Frise	25 (4.4%)	93 (4.3%)	1.00	0.62–1.61		
Miniature Schnauzer	25 (4.4%)	91 (4.2%)	1.02	0.63–1.65		
CKCS	22 (3.9%)	85 (3.9%)	0.96	0.58–1.59		
Cocker Spaniel	20 (3.5%)	79 (3.6%)	0.94	0.56–1.59		
SBT	20 (3.5%)	76 (3.5%)	0.98	0.58–1.65		
Border Collie	17 (3.0%)	64 (2.9%)	0.99	0.56–1.74		
Tibetan Terrier	15 (2.7%)	56 (2.6%)	0.99	0.55–1.81		
Cairn Terrier	11 (2.0%)	47 (2.2%)	0.87	0.44–1.72		
Sex	*n* = 564	*n* = 2176				
Female	268 (47.4%)	1042 (47.8%)	Base			0.876
Male	296 (52.4%)	1134 (52.0%)	1.01	0.84–1.22		
Sex–neuter						
Male entire	13 (2.3%)	139 (6.4%)	Base			<0.001
Female entire	59 (10.4%)	152 (7.0%)	4.15	2.18–7.90	<0.001	
Female neutered	34 (6.0%)	251 (11.5%)	1.45	0.74–2.84	0.280	
Female unknown	173 (30.6%)	638 (29.3%)	2.90	1.60–5.24	<0.001	
Male neutered	24 (4.3%)	247 (11.3%)	1.04	0.51–2.11	0.916	
Male unknown	262 (46.4%)	750 (34.4%)	3.74	2.08–6.71	<0.001	
Sex unknown	0 (0.0%)	2 (0.09%)	–	–	–	
Insured						
Not insured	337 (59.7%)	1848 (84.8%)	Base			<0.001
Insured	228 (40.4%)	331 (15.2%)	3.77	3.08–4.64		
Veterinary group						
D	278 (49.2%)	453 (20.8%)	Base			<0.001
A	5 (0.6%)	11 (0.5%)	0.74	0.25–2.15	0.582	
B	189 (33.4%)	604 (27.7%)	0.51	0.41–0.64	<0.001	
C	85 (15.0%)	122 (5.6%)	1.14	0.83–1.56	<0.001	
E	8 (1.4%)	989 (45.4%)	0.01	0.01–0.03	<0.001	

*Note*: Descriptive statistics and univariable logistic regression for variables associated with developing diabetes mellitus in dogs 4 years or older attending UK primary‐care practices within VetCompass in 2016–2017 (*n* = 2744).

Abbreviations: AB, antibiotic; CI, confidence interval; CKCS, Cavalier King Charles Spaniel; JRT, Jack Russell Terrier; SBT, Staffordshire Bull Terrier; WHWT, West Highland White Terrier.

^a^ Wilcoxon rank sum test.

At the index date, dogs diagnosed with DM had a median age of 10.0 years (IQR 8.4–11.5, range 4.7–15.4), 47.4% (268/565) were female and 40.4% (228/565) were insured (Table 1). At the index date, control dogs had a median age of 8.3 years (IQR 6.1–11.3, range 4.1–21.0), 47.8% (1042/2179) were female and 15.2% (331/2179) were insured. The median age at which control dogs were last known not to have DM was 10.4 years (IQR 8.3–12.9, range 4.1–21.0).

### Descriptive statistics

Exposure to glucocorticoids within the 6 weeks prior to the index date was documented in 9.0% (51/565) of cases and 2.7% (58/2179) of controls (*p* < 0.001). Proportional use of glucocorticoids varied widely between breeds (Table 2). The breeds most commonly exposed to glucocorticoids were West Highland White Terriers (20.7% [19/92] cases and 6.8% [25/370] controls), Tibetan Terriers (20.0% [3/15] cases and 3.6% [2/56] controls) and Cavalier King Charles Spaniels (13.6% [3/22] cases and 3.5% [3/85] controls). Breeds with no reported exposure to glucocorticoids included Cairn Terriers and Cocker Spaniels for cases and Border Collies for both cases and controls. Prednisolone was the most frequently prescribed glucocorticoid, prescribed in 96.1% of cases receiving a glucocorticoid (49/51) and 74.1% of controls (43/58). Disorders of the skin or ears were the most common indication for prescribing glucocorticoids overall, accounting for 70.6% (36/51) of cases and 65.5% (38/58) of controls.

**TABLE 2:** Descriptive statistics for glucocorticoid exposure for dogs developing diabetes mellitus and breed frequency matched controls (*n* = 2744).

Glucocorticoid exposure	Cases (*n* = 565)	Controls (*n* = 2179)
All dogs	9.0% (51/565)	2.7% (58/2179)
By breed		
WHWT	20.7% (19/92)	6.8% (25/370)
Tibetan Terrier	20.0% (3/15)	3.6% (2/56)
CKCS	13.6% (3/22)	3.5% (3/85)
Miniature Schnauzer	12.0% (3/25)	1.1% (1/91)
Yorkshire Terrier	10.2% (5/49)	1.1% (2/189)
Labrador Retriever	10.0% (4/40)	1.3% (2/159)
JRT	8.8% (5/141)	2.2% (5/224)
Bichon Frise	8.0% (2/25)	1.1% (1/93)
SBT	5.0% (1/20)	2.6% (2/76)
Crossbreed	3.6% (5/141)	2.1% (11/523)
Border Terrier	3.2% (1/31)	1.6% (2/123)
Cairn Terrier	0.0% (0/11)	2.1% (1/47)
Cocker Spaniel	0.0% (0/20)	1.3% (1/79)
Border Collie	0.0% (0/17)	0.0% (0/64)
By age group		
4 to <7 years	5.0% (3/60)	1.9% (15/809)
7 to <9 years	9.9% (12/128)	2.8% (13/472)
9 to <12 years	9.1% (25/276)	2.8% (15/529)
12+ years	10.9% (11/101)	4.1% (15/369)
By sex–neuter status		
Female entire	6.8% (4/59)	4.0% (6/152)
Female neutered	5.9% (2/34)	2.8% (7/251)
Female unknown	10.4% (18/173)	2.2% (14/638)
Male entire	7.7% (1/13)	2.9% (4/139)
Male neutered	8.3% (2/24)	4.1% (10/247)
Male unknown	9.2% (24/262)	2.3% (17/750)
Sex unknown	–	0.0% (0/2)
Indication for prescribing	Cases (*n* = 51)	Controls (*n* = 58)
Skin/otitis	70.6% (36)	65.5% (38)
Respiratory	7.8% (4)	12.1% (7)
Gastrointestinal	5.9% (3)	3.4% (2)
Neurological	3.9% (2)	8.6% (5)
Mass	2.0% (1)	5.2% (3)
Miscellaneous	9.8% (5)	5.2% (3)
Duration of course		
Long term (>14 days)	70.6% (36)	77.6% (45)
Medium term (7–14 days)	19.6% (10)	8.6% (5)
Short term (<7 days)	9.8% (5)	13.8% (8)
Type of glucocorticoid		
Prednisolone	96.1% (49)	74.1% (43)
Dexamethasone sodium phosphate	3.9% (2)	8.6% (5)
Methylprednisolone	0% (0)	12.1% (7)
Methylprednisolone acetate	0% (0)	5.2% (3)

*Note*: Descriptive statistics for glucocorticoid exposure within 6 weeks prior to the index date for dogs 4 years or older developing diabetes mellitus, and breed frequency matched controls, who attended UK primary‐care practices within VetCompass in 2016–2017 (*n* = 2744).

Abbreviations: CKCS, Cavalier King Charles Spaniel; JRT, Jack Russell Terrier; SBT, Staffordshire Bull Terrier; WHWT, West Highland White Terrier.

There were 2876 unique antibiotic courses recorded prior to the index date in all dogs under analysis. Dogs prescribed at least one unique antibiotic course accounted for 39.3% of all cases (222/565) and 41.4% of controls (902/2179) (Table 3), and varied between breeds and cases and controls. Miniature Schnauzers (64.0% [16/25] cases and 42.9% [39/91] controls) and Bichon Frise (56.0% [14/25] cases and 40.9% [38/93] controls) breeds contained a high percentage of dogs having at least one unique antibiotic course documented, whereas Jack Russell Terriers (15.6% [22/141] cases and 35.3% [79/224] controls) and Border Collies (17.6% [3/17] cases and 46.9% [30/64] controls) contained a low percentage.

**TABLE 3:** Descriptive statistics for the unique antibiotic (AB) courses prescribed to dogs developing diabetes mellitus and breed frequency matched controls (*n* = 2744).

	Cases (*n* = 565)	Controls (*n* = 2179)
AB courses		
Number of unique AB courses	659	2217
Number of unique AB courses per year of documented dog history		
Median (range)	0 (0–5.26)	0 (0–10.44)
Interquartile range	0–0.61	0–0.48
Number dogs having at least one unique AB course		
All dogs	39.3% (222/565)	41.4% (902/2179)
By breed		
Miniature Schnauzer	64.0% (16/25)	42.9% (39/91)
Bichon Frise	56.0% (14/25)	40.9% (38/93)
Cairn Terrier	54.5% (6/11)	42.6% (20/47)
SBT	45.0% (9/20)	39.5% (30/76)
WHWT	44.6% (41/92)	45.4% (168/370)
Border Terrier	41.9% (13/31)	44.7% (55/123)
Tibetan Terrier	40.0% (6/15)	50.0% (28/56)
Yorkshire Terrier	36.7% (18/49)	39.2% (74/189)
CKCS	36.4% (8/22)	43.5% (37/85)
Crossbreed	36.2% (51/141)	37.1% (194/523)
Cocker Spaniel	30.0% (6/20)	54.4% (43/79)
Labrador Retriever	22.5% (9/40)	42.1% (67/159)
Border Collie	17.6% (3/17)	46.9% (30/64)
JRT	15.6% (22/141)	35.3% (79/224)
Type of AB		
CIA class of unique AB course	*n* = 659	*n* = 2217
Highest priority critically important	5.3% (35)	6.5% (143)
High priority critically important	65.4% (431)	54.6% (1211)
Top six AB prescribed		
Clavulanate potentiated amoxicillin	50.1% (330)	42.9% (951)
Cephalexin	18.2% (120)	15.8% (351)
Metronidazole	10.8% (71)	9.9% (220)
Amoxicillin	4.2% (28)	11.6% (257)
Clindamycin	5.6% (37)	7.6% (169)
Cefovecin	3.2% (21)	2.8% (63)

*Note*: Descriptive statistics for the unique AB courses prescribed to dogs 4 years or older developing diabetes mellitus, and breed frequency matched controls, who attended UK primary‐care practices within VetCompass in 2016–2017 (*n* = 2744).

Abbreviations: CIA, critically important antimicrobials; CKCS, Cavalier King Charles Spaniel; JRT, Jack Russell Terrier; SBT, Staffordshire Bull Terrier; WHWT, West Highland White Terrier.

Antibiotics classified by the WHO as highest priority critically important (HPCIA) accounted for 5.3% (35/659) of all prescribed courses in cases and 6.5% (143/2217) in controls. Clavulanate‐potentiated amoxicillin was the most commonly prescribed antibiotic overall, accounting for 50.1% (330/659) of all courses prescribed to cases and 42.9% (951/2217) of courses prescribed to controls. The number of unique antibiotic courses prescribed per year of documented dog history was positively skewed, with a median of zero for both cases (range 0–5.3) and controls (range 0–10.4).

### Univariable logistic regression

The results of univariable logistic regression on data from all dogs are described in Table 1. All of the variables, except sex on its own, were associated with the diagnosis of DM at the *p*‐value less than 0.2 cut‐off level to take forward to multivariable logistic regression.

Univariable logistic regression on all dogs, when compared to that on the subset dogs with at least 1 year of documented history, yielded variable p‐values that did not differ accross the p‐value cut‐off for taking forwards to multivariable logistic regression. Additionally, the OR for individual categories did not vary by more than 10% between the models, except for the odds of DM in FE dogs compared to ME dogs (all dogs: OR 4.15, 95% CI 2.18–7.90, *p* < 0.001; dogs with 1 or more year of history: OR 2.98, 95% CI 1.40–6.32, *p* = 0.004). As these results did not differ significantly, the final multivariable logistic regression modelling included all dogs.

### Mixed effects multivariable logistic regression

The final multivariable model (Table 4) contained six variables, both main factors of interest (glucocorticoid use within 6 weeks of the index date and total number of unique antibiotic courses documented per dog) along with four confounders: breed, age, sex–neuter status and insurance status. Clustering at the practice level was significant (intraclass correlation coefficient [ICC] = 0.27, 95% CI 0.20–0.36, *p* < 0.001). No biologically plausible interactions were identified. The final model had an acceptable fit (Hosmer–Lemeshow test statistic: *p* = 0.389) and good discrimination (area under the ROC curve: 0.893).

**TABLE 4:** Mixed effects multivariable logistic regression results (*n* = 2742).

Variable	Odds ratio	95% CI	Category *p*‐value	Variable *p*‐value
Breed frequency matched				0.283
Total number of unique AB courses documented per dog				
0	Base			0.024
1	0.65	0.46–0.91	0.012	
2	0.89	0.58–1.35	0.585	
3+	1.22	0.83–1.79	0.305	
Glucocorticoid use 6 weeks prior to index date				
No glucocorticoid use	Base			<0.001
Glucocorticoid use	4.07	2.41–6.89		
Age group				
4 to <7 years	Base			<0.001
7 to <9 years	3.44	2.35–5.04	<0.001	
9 to <12 years	8.06	5.62–11.58	<0.001	
12+ years	3.83	2.55–5.76	<0.001	
Sex–neuter				
Male entire	Base			<0.001
Female entire	5.50	2.56–11.82	<0.001	
Female neutered	1.66	0.75–3.63	0.208	
Female unknown	2.09	1.05–4.16	0.037	
Male neutered	1.16	0.50–2.66	0.730	
Male unknown	2.70	1.36–5.33	0.004	
Insured				
Not insured	Base			<0.001
Insured	3.64	2.75–4.80		

*Note*: Mixed effects multivariable logistic regression (clinic as random effect) for risk factors associated with the diagnosis of diabetes mellitus in dogs 4 years or older attending UK primary‐care practices within VetCompass in 2016–2017, frequency matched for breed (*n* = 2742).

Abbreviations: AB, antibiotic; CI, confidence interval.

**FIGURE 1:**
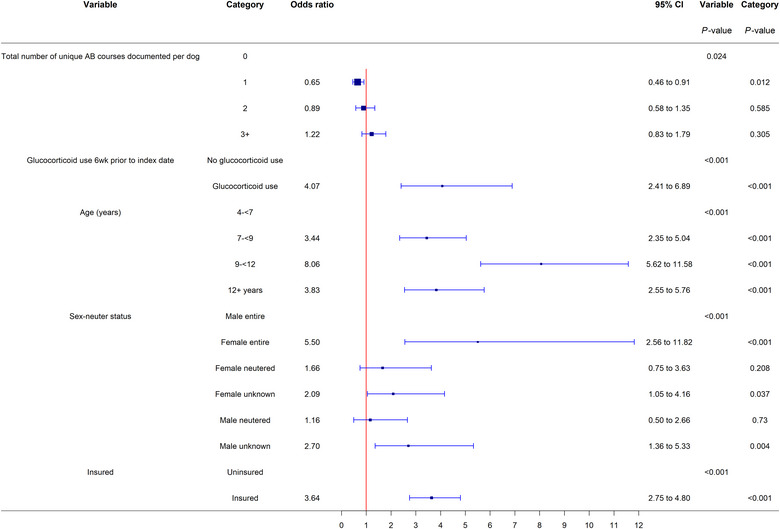
Forest plot of the mixed effects multivariable logistic regression odds ratios with corresponding 95% confidence intervals (CIs) for demographic risk factors associated with the diagnosis of diabetes mellitus in dogs 4 years or older attending UK primary‐care practices within VetCompass in 2016–2017, frequency matched for breed (*n* = 2742). Categories without an odds ratio were the baseline

After adjusting for the effects of the other variables in the model, dogs exposed to glucocorticoids within the 6 weeks prior to the index date had 4.07 (95% CI 2.41–6.89, *p* < 0.001) times the odds of DM compared to dogs that were not exposed to glucocorticoids (Figure 1). Dogs that had one unique antibiotic course documented in their history had a decreased odds of developing DM (OR 0.65, 95% CI 0.46–0.91, *p* = 0.012) compared to dogs that had no documented courses of antibiotics.

The other variables considered in the modelling were included as confounding factors for associations between medication and DM. Among these variables, there were several associations with the development of DM. The odds of DM diagnosis increased with age up to 12 years and then decreased. Compared to dogs aged 4 to less than 7 years, dogs aged 9 to less than 12 years had 8.06 (95% CI 5.62–11.58, *p* < 0.001) times the odds of DM. Compared to ME dogs, FE dogs (OR 5.50, 95% CI 2.56–11.82, *p* < 0.001) had an increased odds of DM. Insured dogs had 3.64 (95% CI 2.75–4.80, *p* < 0.001) times the odds of DM compared to uninsured dogs (Figure 1).

## DISCUSSION

This is the first study to explore the association between the prescribing behaviour of primary‐care clinicians for glucocorticoids and antibiotics and the subsequent risk of dogs developing DM. A major finding of this study is that dogs exposed to glucocorticoids in the 6 weeks prior to the index date had over four times the odds of developing DM. After adjusting for other variables in the final model, risk factors for DM also included dogs 7 years or older, FE dogs and insured dogs. There was some evidence that dogs prescribed one unique course of antibiotics had a lower odds of developing DM.

The 2‐year period prevalence of DM in the current study was 0.38% (95% CI 0.36–0.39) and the 2‐year incidence risk was 0.14% (95% CI 0.13–0.15). This is consistent with the prevalence reported by other primary‐care DM studies (0.32–0.36%).^1^, ^3^, ^4^ Only one study^79^ reported an incidence rate of 13 cases per 10,000 dog years at risk, but this study was restricted to insured dogs, making comparison of incidence with the current study difficult. Referral centre studies report a higher prevalence at 0.64–1.33%,^19^, ^80^ but it is hard to compare with primary‐care studies because of referral bias towards sick animals.^81^

### Glucocorticoid exposure

The current study showed that dogs treated at least once with systemic glucocorticoids within the preceding 6 weeks had over four times the odds of developing DM compared to non‐diabetic control dogs.

Glucocorticoid therapy causes a number of metabolic side effects, including hyperglycaemia and insulin resistance,^36^, ^37^, ^82^ and has been associated with DM in humans.^29^, ^83^, ^84^, ^85^, ^86^ An association between glucocorticoid therapy and DM has also been documented in cats.^87^, ^88^, ^89^ However, the link between glucocorticoid therapy, glucose metabolism and DM in dogs is more controversial, and there have been few studies reporting an association between glucocorticoid exposure and DM in dogs.

Case studies have reported glucocorticoid‐associated hyperglycaemia and DM in dogs,^33^, ^34^ but these were individual cases, and being derived from referral populations, they may be poorly representative of the general dog population. Studies in healthy laboratory Beagle dogs have also shown that oral prednisone can cause hyperglycaemia at higher doses (4 mg/kg daily),^90^ but anti‐inflammatory doses of prednisone^91^ and prednisolone^92^, ^93^ did not result in increased blood glucose concentrations. In atopic dogs of various breeds, anti‐inflammatory doses of oral prednisolone have been shown to increase serum insulin levels after 6 weeks of treatment, although no increase was detected in serum fructosamine, suggesting that serum glucose levels were not significantly raised.^94^ Again, these studies were small in size and limited to specific breeds or disease conditions, so they may not be generalisable to a wider dog population. It is likely that the overall effects of glucocorticoids on DM risk are complex and highly dependent on the duration, dosage and susceptibility of the individual.^29^

In a previous study from the current research team on a population of dogs containing all breeds under primary veterinary care, we reported that diabetic dogs had over two times the odds of having been exposed to systemic glucocorticoids within the preceding 6 weeks before diagnosis compared to non‐diabetics (OR 2.19, 95% CI 1.02–4.70, *p* = 0.044).^2^ Compared to this, the current study contained a larger number of incident cases (687 compared to 409) but included only selected breeds with 10 or more DM cases. In these breeds, both common breeds and those with a high intrinsic risk of DM, exposure to glucocorticoids was associated with DM with an OR of 4.07 (95% CI 2.41–6.89, *p* < 0.001). This leads to the hypothesis that the association between glucocorticoid use and DM may be driven directly by genetic variants present in particular breeds or indirectly by other factors, such as breed predisposition to diseases requiring glucocorticoid therapy or the type and duration of glucocorticoids frequently prescribed. Further research into breed‐related associations between glucocorticoids and DM may help to explain the variability in both differing risk factors and pathogenesis of DM between different breeds.

### Antibiotic exposure

Over 40% (41.0%) of dogs in this study had evidence of exposure to at least one prescription of systemic antibiotics in their documented history. HPCIA^76^ accounted for 6.2% (95% CI 5.3–7.1) of all prescriptions, and the most frequently prescribed antibiotic was clavulanate‐potentiated amoxicillin, in 44.5% (95% CI 43.7–47.3) of all prescriptions, both of which are consistent with other UK studies.^65^, ^69^ Cefovecin, a third‐generation cephalosporin classed as an HPCIA, accounted for 2.9% (95% CI 2.3–3.5) of all prescriptions. This is higher than the previously reported 1.3%^65^ and 1.5%^69^ and may reflect the older population of dogs, the specific breeds included in this study or a change in prescribing behaviour.

There was weak evidence of some association between the number of unique courses of antibiotics documented per dog and the development of DM (*p* = 0.024), but when compared to no documented courses, only one documented course of antibiotic was significant at a *p*‐value less than 0.05. Interestingly, this showed a protective effect, with dogs receiving one unique course of antibiotic having only 0.65 times the odds of DM compared to dogs receiving no documented courses (OR 0.65, 95% CI 0.46–0.91, *p* = 0.012). Research using rodent models of DM has suggested that antibiotic treatment targeting Gram‐negative bacteria in the microbiota can protect against DM, with this effect most prominent when given in utero.^52^ It is not fully understood how the interactions and alterations in microbiota composition may contribute to the development of DM, but antibiotic modulation of this process may well have both positive and negative effects. Although one possibility is that one course of treatment is associated with protection against DM in dogs, it is very difficult to determine in this current study if it is a true association or a chance result.

There are a number of limitations of using EPRs to determine antibiotic exposure prior to a specific event, the most challenging being the length and completeness of documented history for each patient. The median length of documented history prior to the index date was not significantly different between cases (median 1.74 years) and controls (median 2.16 years), but the range between individual dogs was wide (cases: 0–11.32 years; controls: 0–17.14 years). The lifetime exposure to antibiotics for each dog may not have been accurately represented by the history available for analysis. In an attempt to minimise the confounding effect of the variable length of history on the number of unique antibiotic courses documented, two approaches were taken. First, a variable counting the number of unique antibiotic courses over a time period of only 1 year prior to the index date was analysed, but this variable was not as good a fit in the final model as the absolute count of unique antibiotic courses. Second, a parallel analysis was performed using a subset of dogs that had at least 1 year of documented history prior to the index date (1855 dogs, 365 cases and 1490 controls). However, the results from this analysis were not significantly different from those containing all dogs, so to increase the power of the study, all dogs were retained for the final model building.

In this study, the antibiotic exposure variable was defined as a count of the number of antibiotic courses prescribed to an individual dog. However, the mechanism of action of antibiotics on DM via the microbiome is complicated, and the spectrum of activity, duration, dose and timing of exposure may all be critical. These represent alternate ways to categorise exposure to antibiotics as a variable, but were not explored in this study. Human and rodent studies on type 1 DM have specifically concentrated on the timing of antibiotic exposure in early life, when the microbiome and immune system are developing alongside each other.^39^, ^42^ Understanding the impact of antibiotic exposure during these early years and the subsequent development of DM, potentially many years later, was outside the scope of this project, but the evidence in the current study of possible associations between antibiotic use and DM does suggest that future studies exploring exposure in this early timeframe are warranted.

### Additional risk factors

Older dogs were strongly associated with an increased risk of DM diagnosis, which is consistent with other studies.^2^, ^19^ Dogs aged 9 to less than 12 years of age had over eight times the odds of DM diagnosis compared to dogs aged between 4 and less than 7 years. The sex–neuter status was also strongly associated with DM diagnosis. The category with the highest OR was FE dogs, who had over five times the odds of DM compared to entire males. It is well reported that hormonal changes during dioestrus are a risk factor for DM,^31^, ^38^, ^95^ but interestingly there was no significant difference detected in the current study between the odds of DM when comparing entire females with any category other than entire males. This may suggest that entire females are at risk, or potentially that entire males are ‘protected’. Although the current study found no increase in risk for neutered males, other studies have found neutered males to have an increased odds of DM when compared to entire males,^2^, ^3^, ^4^, ^19^ and there is evidence from human studies that low testosterone levels are a risk factor for type 2 DM.^96^ It has been suggested that testosterone‐driven effects on the microbiome may be involved in this mechanism,^97^ and may be puberty dependent.^98^ Therefore, age at the time of neutering may be a crucial factor and may confound the results seen in different studies between the association of neutered males and DM.

Insured dogs were associated with twice the odds of DM compared to uninsured dogs. There is mixed evidence for the risk of insurance on DM, either an increased risk^3^ or no change in risk.^2^ DM is a relatively cheap and simple condition to diagnose in primary‐care practice,^6^ so the cost of diagnosis may not fully explain this strong association for insurance. The default status for insurance status on most veterinary computer systems is uninsured. After diagnosis with DM, a condition that can be expensive to treat, it is not unreasonable to assume that insurance status is checked in the majority of cases and updated on the EPR. A partial explanation of the association between insurance and DM diagnosis may therefore be a reflection of this recording bias. Another consideration is that this association may reflect that the owners who insure their dogs are also more likely to seek veterinary attention and a full diagnosis when their dog becomes sick.

### Limitations

Although this study has highlighted associations between some variables and the development of DM, it is important to remember that this does not necessarily imply a causal relationship.

This current study used data from EPRs not recorded primarily for research and, as already discussed, has some limitations. The EPRs rely on practitioners to input information into the data fields; however, some EPRs may be incomplete, and this effect cannot be assumed to happen at random between the cases and controls. To minimise misclassification of glucocorticoid exposure, all records were manually checked. This allowed identification of animals exposed to glucocorticoids within the 6 weeks prior to the index date, even if they were on a long‐term prescription or if the drugs were not charged through the practice management system. This was, however, unfeasible to do with antibiotic prescriptions because all records prior to the index date would need to have been checked, so some prescriptions may have been missed. The sex–neuter variable was also manually checked. While sex status was fairly easy to determine, neuter status was harder to confirm, and 66.5% (1825/2744) of dogs in this study were of unconfirmed neuter status. This limited the power of this study to identify differences in odds between the categories of neutered and entire animals. Dogs either with undiagnosed DM or in a pre‐diabetic state may be more likely to be prescribed antibiotics. To minimise this bias, any antibiotic prescription within the 30 days prior to the index date was discounted. There is also the potential that dogs selected as controls will subsequently develop DM. To minimise this risk, the records of potential control dogs were manually checked up to January 2021, and any dog developing DM was discounted. The median age at the last recorded patient–practice interaction for control dogs was 10.4 years (IQR 8.3–12.9, range 4.1–21.0), which is similar to the median age of diagnosis for case dogs (median 10.0 years, IQR 8.35–11.46, range 4–18.0).

This case–control study frequency matched cases to controls by breed in order to control more tightly for the confounding effect of breed on exposure to glucocorticoids and antibiotics and on the development of DM. However, this means that the results may not be generalisable to all breeds and instead only represent the common breeds and those breeds with a higher intrinsic risk of DM that were included in this study.

## CONCLUSION

This study reports a strong association between glucocorticoid exposure and DM diagnosis in primary‐care practice and identified weak evidence between exposure to one unique course of antibiotics and a reduced odds of DM diagnosis. The substantially increased risk of developing DM after glucocorticoid exposure suggests that caution is needed in prescribing glucocorticoids to entire females and to older (7 years or over) dogs in the breeds analysed in this study. It also highlights the need to further understand the mechanisms involved in the development of DM and to determine whether specific populations are more at risk than others. Further work is also required to explore the association between antibiotic therapy and DM, with consideration given to the timing of exposure and the impact this may have on the developing microbiome.

## AUTHOR CONTRIBUTIONS

Angela M. Heeley, Dan G. O'Neill and Dave C. Brodbelt were responsible mainly for the conception and design, acquisition and extraction of data. Angela M. Heeley carried out the analysis. Angela M. Heeley, Dan G. O'Neill, Lucy J. Davison, David B. Church and Dave C. Brodbelt were involved in interpretation of the results, drafting and revising the manuscript and gave final approval of the version to be published. Angela M. Heeley, Dan G. O'Neill, Lucy J. Davison, David B. Church and Dave C. Brodbelt agree to be accountable for all aspects of the accuracy or integrity of the work.

## CONFLICT OF INTEREST STATEMENT

The authors have no conflicts of interest to declare.

## ETHICS STATEMENT

Ethical approval was granted by the RVC Social Science Ethical Review Board (reference number SR2018‐1652).

## Data Availability

The VetCompass dataset used for this study is available through open access on the RVC data repository.
